# GABAergic neurotransmission and new strategies of neuromodulation to compensate synaptic dysfunction in early stages of Alzheimer’s disease

**DOI:** 10.3389/fncel.2014.00167

**Published:** 2014-06-25

**Authors:** Mauricio O. Nava-Mesa, Lydia Jiménez-Díaz, Javier Yajeya, Juan D. Navarro-Lopez

**Affiliations:** ^1^Neuroscience Research Group, University of RosarioBogotá, Colombia; ^2^Neurophysiology and Behavior Lab, Centro Regional de Investigaciones Biomédicas, School of Medicine of Ciudad Real, University of Castilla-La ManchaCiudad Real, Spain; ^3^Department of Physiology and Pharmacology, University of SalamancaSalamanca, Spain

**Keywords:** septohippocampal system, amyloid-β peptide, excitatory and inhibitory neurotransmission, learning and memory, Alzheimer’s disease

## Abstract

Alzheimer’s disease (AD) is a progressive neurodegenerative disease characterized by cognitive decline, brain atrophy due to neuronal and synapse loss, and formation of two pathological lesions: extracellular amyloid plaques, composed largely of amyloid-beta peptide (Aβ), and neurofibrillary tangles formed by intracellular aggregates of hyperphosphorylated tau protein. Lesions mainly accumulate in brain regions that modulate cognitive functions such as the hippocampus, septum or amygdala. These brain structures have dense reciprocal glutamatergic, cholinergic, and GABAergic connections and their relationships directly affect learning and memory processes, so they have been proposed as highly susceptible regions to suffer damage by Aβ during AD course. Last findings support the emerging concept that soluble Aβ peptides, inducing an initial stage of synaptic dysfunction which probably starts 20–30 years before the clinical onset of AD, can perturb the excitatory–inhibitory balance of neural circuitries. In turn, neurotransmission imbalance will result in altered network activity that might be responsible of cognitive deficits in AD. Therefore, Aβ interactions on neurotransmission systems in memory-related brain regions such as amygdaloid complex, medial septum or hippocampus are critical in cognitive functions and appear as a pivotal target for drug design to improve learning and dysfunctions that manifest with age. Since treatments based on glutamatergic and cholinergic pharmacology in AD have shown limited success, therapies combining modulators of different neurotransmission systems including recent findings regarding the GABAergic system, emerge as a more useful tool for the treatment, and overall prevention, of this dementia. In this review, focused on inhibitory systems, we will analyze pharmacological strategies to compensate neurotransmission imbalance that might be considered as potential therapeutic interventions in AD.

## INTRODUCTION

Along last three decades, dementias are becoming a worldwide epidemiological problem. The importance of understanding the molecular basis of dementias and designing rational therapies for its treatment is of growing interest for populations where life expectancy along with concerns for a better quality of life are increasing. In December 2005, it was estimated that there were 24.3 million people living with dementia, there would be 31 million in 2010, and people affected by dementia will double every 20 years, rising to 81.1 million in 2040. But the reality is even worse than those approximations. People with dementia need a great amount of support and care that imply a high cost in terms of emotional, social, and financial resources that are mainly provided by their families with the help of local governments or insurance companies. Being ailments that run over a significant time period, the direct and indirect cost of medical care, employment of domestic caregivers, lost productivity in the immediate family, etc., is enormous. A joint effort from researches and health authorities needs to be made to deepen understanding of the etiology and physiopathology of these diseases and therefore develop therapies that improve health and welfare of people with dementia.

Alzheimer’s disease (AD) is the most prevalent cause of dementia among more than a hundred dementia types, and is the major cause of dementia in the elderly (around 50% for age range of 80–89 years old). According to the World Alzheimer Report 2010–2012 (Alzheimer’s Disease International), in 2010 there were about 36 million cases of AD and other dementias in the world, which will increase to 115.4 million in 2050. AD is a devastating progressive neurodegenerative disease characterized by cognitive decline, brain atrophy due to neuronal and synapse loss, and two neuropathological lesions firstly described by Alois Alzheimer in 1907: extracellular amyloid plaques and neurofibrillary tangles formation, composed of amyloid-beta peptide (Aβ) and intracellular aggregates of hyperphosphorylated tau protein, respectively (Goedert and Spillantini, 2006). Together with Parkinson’s disease, Huntington’s disease, transmissible spongiform encephalopathies and amyotrophic lateral sclerosis, AD is one of the neurodegenerative diseases that presents a pathological common mechanism (Soto, 2003) consistent on conformational disorders of a particular protein which can fold into a stable alternative conformation. In most cases, this alteration results in its aggregation and accumulation in tissues as fibrillar deposits that finally induce neuronal death (Bucciantini et al., 2002).

The events that trigger the main pathological changes in AD take place in regions of the temporal lobe, including the medial septum, hippocampus, amygdala, and entorhinal cortex. The early onset of AD is manifested as an inability to form new memories. However, the multiple structural and biochemical changes which are already documented in the mid-to-late stages of AD (such as synapse loss, plaque accumulation, tangle formation, and neurodegeneration) do not explain the memory deficits observed in the early stages of the illness (Selkoe, 2002). For example, the loss of synapses appears to be the best morphological correlate for functional deficits observed in the middle and late stages of AD, but many patients in early stages do not show a significant decline in number of synapses (Terry, 2004; Kelly et al., 2005; Spires-Jones and Knafo, 2012).

Based on these findings, attempts have been made to find an explanation for cognitive deficits observed at early stages of the disease when no significant decline in the synapse and cell number has been detected. It has been proposed that misfolded oligomeric forms or small Aβ aggregates that are not deposited in the tissue might induce an initial state of *synaptic dysfunction* in early AD patients. Numerous genetic, biochemical, and animal model studies have implicated the gradual contribution of Aβ, as a medium for AD. In this sense, it has also been suggested that insoluble amyloid plaques would also have a pathogenic role serving as relatively inert reservoirs of soluble toxic Aβ aggregates that could readily be activated and disassembled by exposure to biological lipids (Martins et al., 2008). This synaptic dysfunction scenario could explain the cognitive deficits observed in the early stages of AD and, thus, precede synapse loss, plaque accumulation, tangle formation, and neurodegeneration (Klein, 2002; Selkoe, 2002; Soto, 2003). However, the mechanisms underlying functional deficits are not known yet.

During the last decade it has been suggested that an imbalance between excitatory and inhibitory neurotransmission systems might underlie the synaptic dysfunction caused by Aβ (Palop et al., 2007; Sun et al., 2009; Palop and Mucke, 2010a; Verret et al., 2012). Pharmacological treatments based on modulating excitatory and/or inhibitory neurotransmission have shown to improve AD symptoms (Farlow, 2009; McKeage, 2009), so that strategies aimed to reestablish the balance between both systems, particularly in early stages of the disease, seem to be the most appropriate to act on the functional deficits caused by Aβ (Huang and Mucke, 2012; Mucke and Selkoe, 2012; Verret et al., 2012).

In this regard, the present paper will review the state of the art of Aβ interactions on excitatory and mainly inhibitory neurotransmission in memory-related brain systems such as amygdaloid complex and septohippocampal system. These regions have shown to be critical in cognitive functions and their neurotransmission systems, particularly the inhibitory one, emerge as pivotal targets for drug design studies to improve learning processes and cognitive dysfunctions that manifest with age.

## Aβ AND EXCITATORY NEUROTRANSMISSION

Several hypotheses have been postulated to explain the neurotoxicity of soluble Aβ aggregates on excitatory neurotransmission systems. Some of these proposals include a cascade of reactions that could involve the blockade of the glutamate recruitment by microglia (Hickman et al., 2008), alteration of the glutamatergic neurotransmission (Ashenafi et al., 2005; Santos-Torres et al., 2007), or modification of both glutamate N-methyl-D-aspartate (NMDA) and/or α-amino-3-hydroxy-5-methyl-4-isoxazolepropionic acid/Kainate (AMPA/Kainate) receptors endocytosis process (Hsieh et al., 2006; Uemura et al., 2007). Other authors consider intracellular calcium increase as the neurotoxic mechanism (Rovira et al., 2002; Resende et al., 2007). Data from Gu et al. (2003) support the cholinergic AD theory and therefore affectation of muscarinic receptors (Kar et al., 1996), suggesting alterations of potassium channels as Aβ action mechanism (Zhang and Yang, 2006). None of these possible mechanisms have completely been discarded at the moment.

The hypothesis explaining Aβ neurotoxic effects through actions on glutamatergic receptors have received important supports. Neuroprotection against Aβ toxic effects has been described by NMDA receptor blockade with MK801. This result supports the idea that a persistent hyperpolarization can reduce the Aβ neurotoxicity due to inactivation of NMDA receptors (Harkany et al., 1999). In accordance, reduction of clinical deterioration in the initial AD phases has also been described using the NMDA glutamatergic non-competitive antagonist *memantine* (O’Mahony et al., 1998). Depression of glutamatergic response by Aβ perfusion has been reported using electrophysiological recordings in amygdala and septum (Ashenafi et al., 2005; Santos-Torres et al., 2007). Other authors support that Aβ alters glutamatergic transmission affecting both metabotropic (Shankar et al., 2008; Um et al., 2013) or AMPA/Kainate receptors endocytosis (Hsieh et al., 2006). It has also been proposed that Aβ effect would be mediated by increased NMDA receptors endocytosis (Uemura et al., 2007) and other authors even postulate that Aβ acting on metabotropic receptors (mGluRI) interferes with the regulation of GABAergic transmission (Tyszkiewicz and Yan, 2005).

On the other hand, results supporting the *cholinergic theory* to explain the mechanisms that underlie AD have also been shown by many authors (Langmead et al., 2008). Muscarinic receptors, specifically M1 subtype, have been broadly related to AD. M1 subtype receptor is widely distributed in the brain and is expressed postsynaptically in cortex and hippocampus (Levey et al., 1991, 1995), important areas for learning and memory. *In vitro* studies have demonstrated that activation of muscarinic receptors induces an alternative pathway for amyloid precursor protein (APP) processing which increases secretion of APP soluble fraction and then reduces Aβ toxicity (Fisher et al., 2002). In particular, selective M1 agonist, AF267B, attenuates the major hallmarks of AD and reverses deficits in cognition (Caccamo et al., 2006, 2009). However, recent results suggest that a decrease in I_M_ (the potassium current activated by muscarinic receptor stimulation) may be an integral part of AD pathophysiology (Leao et al., 2012; Duran-Gonzalez et al., 2013), explaining why I_M_ blockers fail to improve cognition in AD clinical trials (Rockwood et al., 1997). Evidence also points out that the initial injurious effects of the fragment of Aβ, Aβ_1__-__42_, on M1 muscarinic receptor-mediated transmission is due to compromised coupling of the receptor with G_q/11_ G-protein (Janickova et al., 2013). Nicotinergic neurotransmission has also been involved in AD early stages, not only through an activation of presynaptic α7-nicotinic acetylcholine receptors (α7-nAChR; Dougherty et al., 2003) but also by interaction with GABAergic (Spencer et al., 2006) and glutamatergic (Wang et al., 2009) systems.

Then in synaptic dysfunction processes, Aβ has been found to present differential effects on AMPA and NMDA receptors. NMDA has been related to Aβ neurotoxicity phenomena. However, cholinergic disruption induced by Aβ can be established at different levels, including cholinergic neurodegeneration, alterations in acetylcholine release, direct modulation of muscarinic receptors and associated effectors, or nicotinergic system.

### GLIAL CELLS IN THE PHYSIOPATHOLOGY OF AD

Recent reports support the new concept that cognitive function arises from a cooperative activity between both neurons and glia (Perea et al., 2009; Fields et al., 2014). This neuron–glia network integrates information and controls synaptic transmission and plasticity in an active way. The term “tripartite synapse” was proposed in order to describe this cellular configuration which involves presynaptic neuron, postsynaptic neuron, and astrocytes (Araque et al., 1999; Perea et al., 2009). Astrocyte-induced neuromodulation has been described in particular brain structures such as hippocampus (Araque et al., 1998; Jourdain et al., 2007), cortex (Ding et al., 2007), and hypothalamus (Gordon et al., 2005). Several gliotransmitters released from astrocytes modulate synaptic plasticity in those brain structures (Yang et al., 2003; Pascual et al., 2005; Panatier et al., 2006) and participate in learning and memory processes.

Disruption of astrocytic functions and therefore in gliotransmission may underline several brain disorders (i.e., depression, schizophrenia, and epilepsy; Rajkowska et al., 1999; Cohen-Gadol et al., 2004; Fellin et al., 2004; Webster et al., 2005), as well as specific neurodegenerative diseases (i.e., parkinsonism; Forman et al., 2005; Halassa et al., 2007). In fact, enhanced astrocytic *tau* expression in aged transgenic animals results in glutamate-transporter activity reduction and consequent neurodegeneration (Komori, 1999; Dabir et al., 2004). In the context of AD, Aβ can disrupt both astrocytic calcium signaling and glutamate uptake capacity (Vincent et al., 2010; Matos et al., 2012). It has been recently shown in an *ex vivo* astrocyte preparation, that Aβ_1__-__42_ reduces the expression of the two major glutamate transporters in astroglia, GLT-1 and GLAST, through Adenosine A2A receptors (de Vivo et al., 2010; Matos et al., 2012). Taking into account that glutamate transporters are necessary for the clearance of excitatory neurotransmitters, the resulting excitotoxic neuronal damage induced by higher levels of Aβ through this mechanism is reasonable in AD. On the other hand, the neuromodulatory function of astrocytes in particular brain structures may explain specific vulnerability to excitotoxicity and neurodegeneration.

Aβ pathological increases induce multiple glial morphological changes. In fact, astrocytes and microglia become activated close to senile plaques in order to internalize and degrade Aβ (Mohamed and Posse de, 2011). Oxidative stress and inflammatory response induced by astrocytes and microglia activation may have a dual role in pathophysiology of AD with neuroprotective and detrimental consequences (Maccioni et al., 2001; Schipper et al., 2006). This is the basis of the therapeutic use of non-steroidal anti-inflammatory drugs (NSAID) in order to delay AD onset, as well as to reduce the rate of disease progression (Cudaback et al., 2014). Unfortunately, no clinical trials are available to support and recommend its use to prevent AD. However, a novel compound (CHF5074) with both anti-inflammatory and gamma-secretase (an enzyme involved in APP processing) modulatory activities in animal models may have a possible therapeutic role to prevent AD (Calza et al., 2013).

As mentioned previously, AMPA and NMDA receptors have been widely implicated in the physiopathology of AD (for review Parameshwaran et al., 2008). Several studies reported that astrocytes express functional NMDA receptors (Kommers et al., 2002; Lalo et al., 2006; Verkhratsky and Kirchhoff, 2007) which are involved in neuronal–glial signaling, synaptic transmission and cerebral vasodilation (Lalo et al., 2006; Palygin et al., 2010; Parfenova et al., 2012). Therefore, Aβ-induced dysfunction of glutamate receptors might affect NMDA receptors expressed in glial cells and, as a consequence, disrupt neuron–glial signal transmission (Mota et al., 2014). NMDA receptor antagonists, MK801 and *memantine*, might attenuate glutamate mediated cell excitotoxicity by excessive stimulation of NMDA receptors in astrocytes and neurons (Lee et al., 2010). In addition, regarding differences between glial and neuronal NMDA receptors, a new selective antagonist (UBP141) of astroglial NMDA receptors with potential therapeutic role in neurodegenerative diseases has been developed (Palygin et al., 2011). Finally, Talantova et al. (2013) showed that Aβ was able to induce astrocytic glutamate release which led to extrasynaptic NMDA receptor activation. In this case, *nitromemantine*, improved NMDA receptor antagonist which selectively inhibits extrasynaptic over physiological synaptic NMDA receptors activity, may protect against Aβ-induced synaptic dysfunction in hippocampus through selective extrasynaptic NMDA receptors blocking. In addition, Aβ has been shown to disrupt gliotransmission by enhancing calcium signaling through astrocytic α7-nAChRs which could as well underlie glial-based AD pathology (Lee et al., 2014).

Thus, devolvement of novel drugs targeting glial signaling may have a possible therapeutic role in AD. In fact, antiepileptic drugs such as *levetiracetam* reversed synaptic dysfunction and learning and memory deficits in human APP (hAPP) transgenic mice (Sanchez et al., 2012). One of the action mechanisms of *levetiracetam* is glutamate and GABA transporters increase in neurons and astrocytes (Ueda et al., 2007). Reduction in glutamate excitotoxicity and enhancement of inhibitory neurotransmission after chronic *levetiracetam* administration demonstrates a molecular mechanism which involves glial cells, to attenuate cognitive abnormalities in AD.

Finally, astrocytes and neurons work together through several metabolic pathways in order to perform new synthesis of glutamate and GABA (Bak et al., 2006). At inhibitory synapses this pathway is called the GABA–glutamine cycle and it depends on GABA transporters and a multi-enzyme machinery that coordinates this process (i.e., GABA transaminase, glutamate decarboxylase, and glutamine synthetase; Bak et al., 2006; Hertz, 2013). Several studies indicate that the activity of glutamine synthetase is decreased in AD. Dysfunction of astrocyte metabolism and therefore glutamate and GABA–glutamine cycles may underlie cognitive impairment in AD (Le Prince et al., 1995; Robinson, 2000; Nilsen et al., 2014). Drugs targeting GABA-metabolizing enzyme and neurotransmitter transporters are of therapeutical interest in GABA-related neurological disorders (Sarup et al., 2003). However, taking into account the different functional roles of glial and neuronal neurotransmitter transporters and the overlapping in GABA/glutamate metabolic pathways, developing of high selective cell-specific drugs is necessary in order to avoid pharmacological interactions and unpredictable side effects.

In summary, glial cells are dramatically affected in AD. Aβ-induced dysfunction of glutamate receptors (NMDA) in astrocytes disrupts neuron–glial signal transmission. On the other hand, Aβ interaction with cholinergic receptors (i.e., α7-nAChR) and glutamate transporters in glial cells may explain neurotoxicity and selective neurodegeneration. Reducing the activation of astrocytes and microglia is the basis of anti-inflammatory drugs in AD. Finally, metabolism and new synthesis of GABA in glial cells might be an interesting target to selective pharmacological modulation.

### Aβ AND EXCITATORY NEUROTRANSMISSION: AMYGDALOID COMPLEX

Amyloid depositions are found not only in the hippocampus but in other subcortical brain structures. In fact, brain amyloidosis in subjects with higher vulnerability to AD pathology (i.e., individuals with mild cognitive impairment, MCI) includes structures such as parietal association cortices, posterior cingulate, precuneus, amygdale, and caudate (Tosun et al., 2013). Among these brain regions, the number of senile plaques has been reported to be the highest in amygdala (Arriagada et al., 1992). Accordingly, a recent diffusion-tensor imaging study has revealed significant decrease in the relative volume of amygdale in early stage AD subjects (Li et al., 2013). The amygdala has long been known to be vulnerable to Alzheimer-type pathology (Hopper and Vogel, 1976), and it has been described as one of earliest locations to develop Alzheimer pathology in Down syndrome (Mann et al., 1986). AD subjects develop brain pathology similar to that of Down syndrome, including Aβ depositions (Nardone et al., 2006). Asymmetrical neuronal loss in the amygdala ranges from 35% to 70% in AD (Scott et al., 1992; Vereecken et al., 1994). In addition, it has been reported that neuronal loss was more severe in the corticomedial regions than in the basolateral region of the amygdala (Tsuchiya and Kosaka, 1990). The distribution pattern of neuronal loss was similar to that of neurofibrillary tangles instead of the distribution of senile plaques (Tsuchiya and Kosaka, 1990). Severity of amygdala pathology correlates with disease duration in AD (Arriagada et al., 1992), and amygdala pathology has been associated with emotional and memory disturbances (Zald, 2003). Despite the importance that amygdaloid complex seems to have, few studies have investigated how Aβ induces injury and may contribute to underlie the emotional and cognitive symptoms typically observed in AD patients.

Based on the amygdala’s cytoarchitecture its subnuclei can be classified in superficial amygdaloid nuclei, centromedial group, and the basolateral complex (Heimer et al., 1999; Amunts et al., 2005). The basolateral amygdaloid complex is formed by the lateral, basolateral, and basomedial nuclei (Swanson and Petrovich, 1998), and innervated by cortical projections across the external capsule. In the amygdala, the excitatory synaptic activity evoked by stimulation of the external capsule is fundamentally mediated through the action of glutamic acid on AMPA/Kainate and NMDA receptors (Rainnie et al., 1991; Smith and Dudek, 1996). The amplitude of these responses is significantly depressed by Aβ without changes in membrane resistance values, which confirms that Aβ effect is localized at synaptic level (Ashenafi et al., 2005). Specifically, the Aβ effect seems to be located at presynaptic level since it could be prevented by calcicludine or nifedipine, both selective antagonists of presynaptic L-type calcium channels (Ashenafi et al., 2005).

Regarding cholinergic neurotransmission, the magnocellular division of the basal nucleus presents a high density of acetylcholinesterase positive fibers (Amaral and Bassett, 1989). The activation of these fibers generates depolarization of long duration in pyramidal cells, which is blocked by atropine (a competitive muscarinic receptor antagonist; Washburn and Moises, 1992; Moises et al., 1995). Muscarinic agonists, such as carbachol, mimic this type of response in the amygdaloid pyramidal neurons (Washburn and Moises, 1992; Yajeya et al., 2000). This effect is mediated by the closure of potassium channels and/or opening of non-specific cationic channels (Yajeya et al., 1997, 1999, 2000). On the other hand, data from Wang et al. (2000) showed that Aβ_1__-__42_ may block presynaptic α7-nAChR in neurons derived from human brain tissues and neuroblastoma cells. It can be assumed that as a consequence of such blocking, the concentration of calcium in the synaptic terminal diminishes, producing a decrease in the amount of neurotransmitter released when the terminal is activated. Although the existence of these receptors has been verified in presynaptic terminals of the amygdala (Girod et al., 2000), a previous study in our group has shown that α7-nAChR was not involved in the Aβ_25__-__35_ short-term neuromodulatory effects in basolateral amygdaloid complex (Ashenafi et al., 2005). In addition, nicotine stimulates mRNA expression of APP in the amygdala (Gutala et al., 2006). However, its functional implications in amygdaloid complex have not been deeply studied.

### Aβ AND EXCITATORY NEUROTRANSMISSION: SEPTOHIPPOCAMPAL SYSTEM

Septum and hippocampus are structures dense and reciprocally interconnected through fimbria–fornix complex, and are functionally coupled to form the septohippocampal system, which shows a critical involvement in generating certain oscillatory activity, such as *theta* rhythm, necessary for fundamental processes in learning and memory (Stewart and Fox, 1990; Bland and Oddie, 2001; Buzsaki, 2002; Sotty et al., 2003; Colom, 2006; Colom et al., 2010; Rubio et al., 2012). *Theta* oscillation coordinates septohippocampal network and depends on interconnections, which include well known cholinergic and GABAergic components (Lynch et al., 1977; Kohler et al., 1984; Bland and Colom, 1993) and recently described glutamatergic projections (Sotty et al., 2003; Huh et al., 2010).

The initial symptoms of AD involve memory impairment and disorientation (McKhann et al., 1984; Swanberg et al., 2004). Damages found in septum and hippocampus could explain those cognitive deficits (Moreno et al., 2007; Palop et al., 2007; Villette et al., 2010; Rubio et al., 2012). Functional images applied in AD subjects have detected defects in the hippocampal formation, a brain structure where the disease begins (Bland and Colom, 1993; Gonzalez et al., 1995; Harris et al., 1998; Wu and Small, 2006). It has been shown that cholinergic cells of the medial septum/diagonal band of Broca (MS–DBB) and the enzymes necessary for the synthesis of acetylcholine, are particularly susceptible to disturbance, with consequent dysfunction in cognitive processes (Yamaguchi and Kawashima, 2001). Intracerebroventricular (i.c.v.) injection of Aβ_25__-__35_: (i) reduces the activity of acetylcholinesterase in the medial septum, cortex, and hippocampus of rats (Yamaguchi and Kawashima, 2001); (ii) decreases performance in passive avoidance and water maze tests (well established learning and memory tests); (iii) and also reduces neuronal loss and appearance of Aβ deposits in cortex, hippocampus, and caudate nucleus (Maurice et al., 1996). Moreover, the septal injection of different fragments of Aβ (Aβ_18__-__28_, Aβ_25__-__35_, or Aβ_1__-__40_) produces a marked reduction in basal or induced release of acetylcholine (Kar et al., 1996). Inhibition of acetylcholine release is also observed in the hippocampus and cortex in* in vitro* rat brain slices using different Aβ fragments (Kar et al., 1996). It has also been shown that Aβ inhibits some of the effects mediated by acetylcholine through septohippocampal muscarinic receptors (Kar et al., 1996; Santos-Torres et al., 2007). This capability could contribute to the particular Aβ vulnerability of cholinergic neuronal populations. However, the low concentrations (nanomolar) with which such effects are obtained, along with the fact that nervous system cells actively secrete Aβ product, suggest the possibility that this peptide may have physiological activity, acting as a neuromodulator not only on the cholinergic, but also on other neurotransmission systems.

*In vitro* studies in septum slices have found that Aβ induces a deficit in glutamatergic synaptic transmission (Santos-Torres et al., 2007). It has recently been observed *in vivo* that septal glutamatergic neurons are vulnerable to Aβ through excitotoxic mechanisms (Colom et al., 2010). The two characteristics of the *theta* rhythm, frequency and amplitude, are affected by modulation of septal NMDA receptors (Puma and Bizot, 1999; Bland et al., 2007). Furthermore, injection of NMDA antagonists in MS–DBB decreases the amplitude of hippocampal *theta* rhythm (Leung and Shen, 2004). This indicates that glutamatergic MS–DBB circuits are also affected by Aβ and are important for the generation and maintenance of septohippocampal rhythmic activity amplitude at *theta* frequencies (Colom et al., 2010).

Aβ has also shown to induce dysfunction of septal glutamatergic neurons involving muscarinic receptor effectors, the potassium voltage-gate channels, KCNQ (Leao et al., 2012). In this case, Aβ diminishes septal rhythmicity by decreasing KCNQ conductance, which negatively affects hippocampal rhythmogenesis and could underlie the memory loss observed in AD (Leao et al., 2012). In this sense, it has been reported that in the septohippocampal system Aβ reduces not only KCNQ2 subunit expression and then, KCNQ conductance, altering the neuronal excitability but also the expression of the oxidative stress-related genes superoxide dismutase 1 (SOD1), 8-oxoguanine DNA glycosylase (OGG1), and monamine oxidase A (MAOA). This situation leads to a neuronal dysfunction and damage that could not be fixed because of the decreased expression of repairing genes (Duran-Gonzalez et al., 2013).

Recent studies have shown that Aβ binds to α7-nAChR in several brain structures including the hippocampus (Spencer et al., 2006; Soderman et al., 2008). On the other hand, it has been proposed that α7-nAChR is functionally blocked in hippocampal CA1 neurons due to an interaction between the receptor and Aβ (Soderman et al., 2011). The functional consequences of such interaction may lead to impairments in both cognitive function (Soderman et al., 2008) and synaptic plasticity (Soderman et al., 2011). *α* 7-nAChR inhibition has also been explained by sustained increase in presynaptic Ca^2^^+^ evoked by Aβ which may underlie disruption of neuronal signaling via nAChRs in the early stages of AD (Dougherty et al., 2003). Since nicotine is able to induce LTP in CA1 hippocampal region probably due to reducing GABAergic inhibition and therefore, increasing the excitability of pyramidal neurons (Fujii et al., 2000), is plausible a functional interaction between α7-nAChR and GABAergic system. However, hippocampal GABAergic interneurons exposed to high levels of amyloid still presented α7-nAChR-mediated activity (Spencer et al., 2006). On the other hand, α7-nAChR and NMDA glutamatergic receptor activities are impaired in synaptosomes derived from AD postmortem tissue and in presence of high Aβ_1__-__42_ levels (Wang et al., 2000). Hence it will be essential to advance in the knowledge of α7-nAChR as therapeutic target for the treatment of Aβ-induced pathology and AD (Kem, 2000; Chen et al., 2006; Dziewczapolski et al., 2009).

## Aβ AND GABAergic NEUROTRANSMISSION

The regulation of many physiological and cognitive processes is depends on a fine tuning between excitatory and inhibitory systems. In order to maintain neural network stability, GABA, the main inhibitory neurotransmitter in the mammalian central nervous system (Cardinali and Golombek, 1998), is known to regulate excitatory activity preventing neuronal hyperexcitation as well as oscillatory activity and firing rate impairments in neural networks (Oren et al., 2006; Zemankovics et al., 2013).

GABAergic neurons are the principal inhibitory neurons and one of the major local circuit neurons (Moore, 1993) which has been implicated in the regulation of a variety of behavioral functions such as learning and memory (Chapouthier, 1989; Vinogradova et al., 1998). In fact, GABAergic influence is the key to generate rhythmic synchronization of neurons during *theta* and *gamma* activity in different brain regions, contributing to neuronal communication and memory processing (Somogyi and Klausberger, 2005; Gong et al., 2009). Cortical and hippocampal function depends on optimum levels of inhibition (Borhegyi et al., 2004; Kaifosh et al., 2013; Xu et al., 2013; Bissonette et al., 2014) to maintain an adequate synaptic plasticity activity. For a long time, GABAergic neurotransmission has been considered well preserved in AD (Rissman et al., 2007). However, cumulative evidence indicates that changes in GABAergic neurotransmission are involved in the physiopathology of AD and may be very important as a possible target to pharmacological intervention previous to cognitive dysfunction in early AD. Below, we have reviewed the effect of Aβ on inhibitory GABA system in the different brain structures which relate to cognitive deficits in AD.

It has been already discussed in this review that excitatory neurotransmission contributes to the pathogenesis and progression of AD and as a result could serve to disrupt the excitatory/inhibitory balance in brain structures, participating in memory processing and therefore taking part of mechanisms that could explain cognitive dysfunction. It is important to consider that alterations on inhibitory neurotransmission or GABA receptors may also induce a significant impact on brain structures and functions, and might also participate in the dysregulation of the balance between excitatory and inhibitory neurotransmission seen in AD patients (Palop et al., 2007; Palop and Mucke, 2010b; Mucke and Selkoe, 2012). In fact, increased epileptiform activity and non-convulsive seizure induced by Aβ in both, animals models of AD (Palop et al., 2007) and AD in elderly people (Palop and Mucke, 2009, 2010a; De Simone et al., 2010) suggests that disruption of excitoratory/inhibitory balance by Aβ involves different neurotransmitter systems including the GABAergic.

Cognitive deficits in AD are explained by selective vulnerability, neurodegeneration, and loss of function of neuronal populations and neurotransmitter systems in particular brain regions such as hippocampus, septohippocampal system, and amygdala. Acetylcholine-releasing neurons and glutamatergic neurons in basal forebrain and hippocampus, respectively, are particularly vulnerable to Aβ neurotoxic effects (Coyle et al., 1983; Emre et al., 1992; Danysz et al., 2000; Giannakopoulos et al., 2009) while GABAergic neurons are relatively resistant to neurodegeneration in AD (Rissman et al., 2007). Recently, in a mouse model of AD it has been shown that glutamatergic hippocampal terminals decrease after Aβ_1__-__42_ perfusion while no significant changes in GABAergic terminals were observed (Canas et al., 2014). GABAergic synapses are preserved in human AD and APP/PS1 transgenic mice (Mitew et al., 2013). However, Aβ might have indirect effects on the inhibitory GABAergic transmission as a result of the dynamic GABAergic balance modulation of the other two excitatory systems (cholinergic and glutamatergic neurotransmission). It has recently been suggested that the imbalance between excitatory and inhibitory systems underlies the synaptic dysfunction caused by Aβ (Sun et al., 2009; Palop and Mucke, 2010a). As a consequence of relative sparing of GABA_A_ receptors in AD, GABAergic sprouting in cortical and hippocampal networks enhance synaptic inhibition in order to compensate aberrant increases in network excitability (Palop et al., 2007). Hypersynchronous neuronal activity on those networks and Aβ-induced neurological deficits before neurodegeneration can be explained by this mechanism. Increase on glutamic acid decarboxylase (GAD, the rate-limiting enzyme synthesizing GABA) activity and consequently on the tone of the GABAergic system in AD brains has also been reported (Reinikainen et al., 1988). According to previous GABAergic hypothesis, this increase on extracellular GABA levels leads to alterations in neuronal membrane functions, abnormally enhanced synthesis of APP and facilitates neurodegeneration in basal forebrain system (Marczynski, 1998).

Controversially, selective somatostatin/NPY inhibitory interneurons neurodegeneration has been described in the hippocampus of a transgenic presenilin 1 PS1/APP AD model, with preservation of GABAergic mRNA synaptic markers (Ramos et al., 2006). In the same animal model, hyperactive neurons in cortical circuits are linked with a relative decrease in synaptic inhibition rather than increase in excitatory glutamatergic neurotransmission, suggesting impairments in GABAergic function (Busche et al., 2008; Palop and Mucke, 2010a). A recent report also shows that in mice expressing hAPP, the network dysfunction (hypersynchrony and reduced *gamma* oscillatory activity) and memory deficits in AD might arise from inhibitory interneuron deficit (Verret et al., 2012).

In line with the above scenario, therapies aimed at increasing GABAergic activity may reduce network/synaptic dysfunction on brain structures which participate in memory processing in AD subjects. Besides potential benefits of drugs which attenuate the Aβ-induced synaptic dysfunction, several previous studies have shown that selective GABA_A_ receptor agonists (i.e., muscimol) are able to protect against Aβ-induced neurotoxicity in retinal, hippocampal, and cortical neurons in rodents (Gu et al., 2003; Paula-Lima et al., 2003; Louzada et al., 2004; Lee et al., 2005). Some neuroprotective effects of GABA modulators (i.e., *Etazolate*, selective GABA_A_ receptor modulator) could be blocked by GABA_A_ receptor antagonists (Marcade et al., 2008), indicating that these neuroprotective effects were due to GABA_A_ receptor signaling and opening new therapeutic possibilities for AD treatment (Vandevrede et al., 2013). In fact, GABA_A_ receptor agonists to treat age-related cognitive deficits were proposed as a new therapeutical approach in the 11th Alzheimer’s Disease Drug Discovery International Conference (Wolfe, 2010a,b). However, none precise action mechanism has been well described. Stimulation of GABA receptors by *pentobarbital* apparently restores neuronal maturation and neurogenesis in apolipoprotein E (APOE)4 knocking mice (Li et al., 2009). Apolipoprotein E4 is considered as a major genetic risk factor for early onset AD perhaps by accelerating Aβ plaque formation, or by impairing neuron repair (Baum et al., 2000; Lopez, 2011; Seppala et al., 2012). Despite the plausible neuroprotective effects of GABA agonists, the widely described side effects limit its long-term use (Lanctot et al., 2004, 2007). Furthermore, there is evidence of long-term effects of benzodiazepines (GABA_A_ agonists) and their relationship with increased risk of dementia (Gallacher et al., 2012).

Cerebrospinal GABA studies as well as neuroimaging and postmortem studies have been useful to show the relationship between GABA system and AD (Jimenez-Jimenez et al., 1998; Yew et al., 1999; Lanctot et al., 2004). Postmortem autoradiographics and benzodiazepine binding studies of GABA receptors in cortex and hippocampus have been controversial to demonstrate changes in GABA_A_ receptors levels; however, most of them have shown a relative decrease in GABA_A_ receptors expression in frontal, temporal, and parietal cortical regions and limbic structures. GABA_A_ subunits specific susceptibility could explain those dissimilar results. However, alteration in GABA_A_ receptor subunits has shown paradoxical results probably due to compensatory mechanisms which are not well described (Mizukami et al., 1997, 1998; Howell et al., 2000; Rissman et al., 2003, 2007; Iwakiri et al., 2009). It is plausible that some effects on GABA dysfunction in AD induced by Aβ are not necessarily associated with a significant damage on GABA neurons or reduced expression of GABA_A_ receptors, and could be explained by functional GABA_A_ receptor activity changes. In fact, a conventional voltage-clamp study showed that Aβ may increase neuronal excitability by inhibiting GABA-induced Cl^-^ current in the neurons of central nervous system (Sawada and Ichinose, 1996). This result suggests that GABA modulators and agonists can normalize Cl^-^ flux and possibly restore the functional properties and excitability of theses neurons. In a series of elegant studies, the microtransplantation of functional receptors and channels from the human AD brain to frog oocytes showed an amplitude reduction of the currents elicited by GABA application, indicating that receptor-channel function was impaired (Miledi et al., 2004) or resulted from a diminished number of GABA_A_ receptors in the membranes of AD brains (Bernareggi et al., 2007). Finally, GABA currents from AD brains have a faster and less sensitive rate of desensitization than those from control brains (Limon et al., 2011), which was explained by down regulation of α1 and γ2 receptor subunits while a compensatory up-regulation of α2, β1, and γ1 receptor subunits took place. Selective pharmacological modulators of GABA_A_ subunits (i.e., α5-selective inverse agonist) may be effective to increase cognitive performance in memory disorders (Atack, 2010). Age-dependent reduction of GABA currents in AD brain from human postmortem tissue indicates a reduction of principal GABA receptors subunits (Limon et al., 2012). However, one of the major problems when GABAergic drugs are chronically used is the desensitization. It has been suggested that such phenomenon could be removed with phosphatase inhibitors or neurotrophic factors which positively modulate GABA currents (Palma et al., 2005; Limon et al., 2011) and both could be potential therapeutic targets for new AD drugs.

Hence, although some AD models have shown that the GABAergic system is relatively well preserved, Aβ might have indirect effects on GABAergic neurotransmission and induce inhibitory interneuron deficits, which could underlie neuronal hyperexcitability observed in AD. On the other hand, long-term Aβ exposition generates increased GABAergic activity and up/down regulation of specific GABA_A_ subunits, as a compensatory mechanism. The understanding of those acute and chronic differential effects of Aβ on inhibitory systems is a pivotal point to develop novel therapeutical strategies to reduce cognitive impairment in early AD.

### Aβ AND GABAergic NEUROTRANSMISSION: AMYGDALOID COMPLEX

As previously discussed in this review, there is general agreement to state that amygdala participates in emotional behavior processing. Afferent and efferent specific connections of the amygdala with a large variety of cortical and subcortical structures are the basis of cognitive functions and affective behaviors such as stress, defense, escape, pain, motivation, emotional discrimination, learning, and memory (Swanson and Petrovich, 1998; LeDoux, 2000; Gill and Grace, 2013). GABAergic afferents originating from amygdaloid interneurons of the basolateral complex which synapse on pyramidal cells probably modulate its function (McDonald, 1985; Carlsen, 1988; Carlsen and Heimer, 1988; McDonald and Augustine, 1993). In fact, altered signaling in GABAergic systems in amygdala produces impairments in emotional learning and memory tasks (Bolton et al., 2012). Regarding the above scenario, it has been proposed that inhibitory GABAergic activity in the basolateral amygdala cooperates to promote amygdala–hippocampal synchrony involved in emotional memory formation (Bienvenu et al., 2012). Amygdala dysfunction has been related to both AD (Bienvenu et al., 2012) and Aβ pathology in rodents (Devi and Ohno, 2010; Huang et al., 2010). Previously, our group demonstrated that GABA_A_ inhibitory evoked responses decreased in amplitude after Aβ perfusion in basolateral amygdaloid complex through a presynaptic mechanism (Ashenafi et al., 2005). A transgenic mice study has shown that accumulation of Aβ in GABA neurons of the basolateral amygdaloid complex was related to enhanced innate and conditioned fear symptoms and spatial memory deficits (España et al., 2010). These results suggest that Aβ-induced dysfunction of GABAergic activity in key brain structures as amygdala might explain the emotional symptoms in AD such as anxiety and fear, as well as faster cognitive decline in memory processing.

A single neurotransmitter imbalance or Aβ-induced neurotoxic effects in a specific brain nucleus would not explain the pathology which involves whole brain regions and circuits. However, some psychological symptoms in AD subjects have been associated with GABAergic changes (Lanctot et al., 2007) and GABA_A_ agonists have been widely used in the treatment of some behavioral and psychological symptoms of AD, such as aggression and agitation (Lanctot et al., 2004). Aβ-induced changes in GABAergic neurotransmitter system in amygdala would help us to understand the pathophysiology mechanism of emotional symptoms in AD subjects.

### Aβ AND GABAergic NEUROTRANSMISSION: SEPTOHIPPOCAMPAL SYSTEM

The function of septal neurons of the basal forebrain is to modulate hippocampus and neocortex circuits’ activity in order to maintain sensory information and memory processes (Colom, 2006; Colom and Garrido-Sanabria, 2007). GABA neurons have been well described in that region in close proximity to cholinergic neurons in the MS–DBB complex (Kimura et al., 1980; Castañeda et al., 2005). In the same complex, burst-firing GABAergic neurons contribute to hippocampal *theta* rhythm *in vivo* (Sotty et al., 2003). Similarly, inhibitory neurons of the medial septum provide rhythmic drive to the hippocampus independently of intrahippocampal *theta* genesis (Hangya et al., 2009). GABA-containing afferents originating in the septum innervate most of the inhibitory interneurons in the hippocampus, which, in turn, control the activity of large populations of excitatory pyramidal cells (Freund and Antal, 1988). As a result, these septohippocampal GABAergic projections have a main role in modulating electrical rhythmic activity in the hippocampal formation.

*In vivo* experiments in rats have shown that cholinergic and GABAergic neurons from medial septum are involved in generating *theta* rhythmicity in the hippocampus (Simon et al., 2006). Predictive modeling of hippocampal microcircuits has shown that inhibitory interneurons from septum have a main role in spatial memories as well as in the maintenance of *theta* phase precession phenomenon of principal cells (Cutsuridis et al., 2010; Cutsuridis and Hasselmo, 2012). Because *theta* and *gamma* activity play a functional role in memory formation and retrieval (Bastiaansen and Hagoort, 2003; Hasselmo, 2005; Lisman, 2005), disruption of septohippocampal projection might explain cognitive deterioration in neurodegenerative diseases. Actually, several studies have demonstrated the relationship between Aβ-induced pathology and septohippocampal dysfunction as follows. In an interesting study, after single injection of Aβ_1__-__40_ into the medial septum, a significant reduced hippocampal *theta* rhythm was reported, associated with damage on cholinergic and glutamatergic neurons activity controlled by the GABAergic system (Colom et al., 2010). Similarly, Aβ_1__-__42_ injection into the MS–DBB complex preferentially injures septal cholinergic neurons but not inhibitory cells (Harkany et al., 1995). Despite these studies have proven that septal GABAergic neurons are spared after acute Aβ injection in septum, this consideration would not mean that Aβ induced septohippocampal dysfunction is not associated with inhibitory functional changes. By using* in vivo* preparations, it has been proposed that reduction of septal cholinergic and glutamatergic inputs onto GABAergic septal neurons may reduce the population of rhythmically bursting GABAergic neurons and suggest that GABAergic neurons are dysfunctional in Aβ-treated rats (Colom et al., 2010). Similarly, hippocampal Aβ_1__-__40/31__-__35_ injections induce a significant impairment of spatial memory in rats and concomitant reduction in the hippocampal *theta* rhythm (Liu et al., 2013). Aβ effects are associated with GABAergic neurons dysfunction and greatly weakened septal *theta* transmission to the hippocampus rather than interfere with its generation. Therefore, hippocampal Aβ-induced pathology reduces the bursting activity of septohippocampal GABAergic neurons (Villette et al., 2010). As previously stated, these neurons contact with hippocampal GABAergic interneurons, which in turn, control the pyramidal neurons rhythm. Since GABAergic septohippocampal neurons and hippocampal interneurons are relatively spared, these effects are probably due to functional regulation changes rather than neurodegeneration or reduction in GABA receptors expression.

Conversely, a study with a triple-transgenic mouse model of AD (TauPS2APP) revealed a significant neurodegeneration of GABAergic septohippocampal projection neurons as well as GABAergic hippocampal neurons (Loreth et al., 2012). Accordingly, loss of GABAergic septohippocampal axon terminals in the mouse model hAPP has been described (Rubio et al., 2012). However, some of these changes are not caused by neuronal loss. Recently, it has been reported *in vivo* that Aβ injection in dorsal hippocampus induces a selective death of GABAergic neuronal subpopulations projecting to the medial septum (Villette et al., 2012). In this sense, even with differences in the currently available models of AD, GABAergic decline in the septohippocampal projections may explain loss of hippocampus oscillatory activity necessary for learning and memory processes.

Hippocampal *theta* and *gamma* rhythms changes occur during the early stages of AD as a product of excitation–inhibition imbalance (Goutagny and Krantic, 2013). As described above, these changes may be related with Aβ effects on MS–DBB and afferents which in turn modulate hippocampus activity. However, hippocampal formation itself is a particularly vulnerable region to Aβ peptide accumulation (Moreno et al., 2007; Double et al., 2010). As a consequence, hippocampal atrophy and neurodegeneration are common features found in AD (Hof and Morrison, 1991; Cummings and Cole, 2002; Casas et al., 2004). Acute Aβ rise induces neurotoxic damage and synaptic dysfunction affecting GABAergic neurotransmitter system in the hippocampus and associative cortex by numerous mechanisms, affecting their function before neurodegeneration and accumulation of Aβ in senile plaques occurs (Selkoe, 2002; Klingner et al., 2003). In fact, cumulative evidence shows that soluble forms of Aβ can interfere with hippocampal synaptic plasticity responsible for learning and memory processing, inhibit long-term potentiation (LTP), and enhance long-term depression (LTD; Chen et al., 2000; Freir et al., 2001; Shankar et al., 2008; Ondrejcak et al., 2010). Hippocampal LTP in an AD animal model, the transgenic APP/PS1 mice, is larger after perfusion with GABA_A_ receptor antagonist as a product of changes in synaptic protein levels. These data suggest that reduced LTP is associated to an enhanced GABA_A_ receptor-mediated inhibition (Fernandez et al., 2007; Yoshiike et al., 2008). Several studies previously reported cognitive improvement in the presence of chronic systemic treatment with GABA_A_ antagonists (i.e., flumazenil or picrotoxin; Marczynski, 1995, 1998; Fernandez et al., 2007; Yoshiike et al., 2008). According to these authors, those blockers are plausible useful therapeutic agents for age-related loss of cognitive functions in AD animal models. Nevertheless, other studies affirmed that Aβ-impairment effects on LTP appear to be independent of GABA_A_ receptor-mediated synaptic inhibition because perfusion with picrotoxin had no effect on the inhibition of LTP (Raymond et al., 2003). Additionally, GABAergic synaptic transmission onto the hippocampus was affected by i.c.v. injection of Aβ_1__-__40_ and Aβ_1__-__42_ (Cullen et al., 1997) or Aβ_25__-__35_ (Sun and Alkon, 2002). Finally, several studies have reported specific GABAergic interneurons decrease (parvalbumin, calretinin, and somatostatin/NPY immunoreactive neurons) in the hippocampus of the APP/PS1 mice (Ramos et al., 2006; Baglietto-Vargas et al., 2010; Takahashi et al., 2010). Therefore, changes in the hippocampal inhibitory systems could explain the cognitive dysfunction in early stages of AD, but it is not clear whether is a consequence of functional decline in GABAergic neurotransmission, specific interneuron degeneration, or an imbalance in the excitatory/inhibitory activity, or a compound of these factors. Differences in the methodological approach (i.e., neuronal recording, genetically modified animal models, data analysis or *in vitro* vs. *in vivo* studies) may explain these paradoxical results. On the other hand, no clinical trial on GABA_A_ receptor antagonists in AD or MCI has been successful or even performed, and therefore new studies are necessary in order to provide a link between basic science and clinical applications.

In summary, since septohippocampal GABAergic projections as well as intrahippocampal inhibitory interneurons have a main role in hippocampal oscillatory activity, inhibitory neurotransmission is an excellent candidate to be affected by Aβ in AD.

### Aβ AND GABA_**B**_ RECEPTORS

Despite the existence and widespread distribution of the GABA metabotropic type receptors (GABA_B_) in the central nervous system (Emson, 2007), their association with Aβ-induced pathology in AD have not been examined deeply yet. GABA_B_ are coupled to intracellular signal transduction mechanisms via G proteins (Mott and Lewis, 1994; Kaupmann et al., 1998). These channels mediate slow and prolonged synaptic inhibition mainly by postsynaptic G protein-coupled activated inwardly rectifying potassium (GirK) channels (Luscher et al., 1997; Kaupmann et al., 1998). At presynaptic level, GABA_B_ receptors also modulate (rather than generate) rhythmic activity in the MS–DBB (Henderson and Jones, 2005) and therefore have a possible role on memory function. GABA_B_ receptor-mediated inhibition regulates the slow oscillation during *gamma* and *theta* oscillations in the control of cortical network activity (Kohl and Paulsen, 2010). *Gamma* oscillations have been associated with sensory processing (Singer, 1993), memory (Fell et al., 2001; Sederberg et al., 2007), attention (Fries et al., 2001), and finally, with consciousness (Llinas et al., 1998). GABA_A_ and GABA_B_ receptors are essential to learning and memory processes (Lasarge et al., 2009; Heinrichs et al., 2010; Mizoguchi and Yamada, 2011) and their pharmacological modulation affect the cognitive function. Since redistribution of hippocampus-dependent memories to neocortical sites is depending on slow oscillations (Diekelmann and Born, 2010), is plausible a role of GABA_B_ receptors on this cognitive functions (Kohl and Paulsen, 2010). Autoradiography studies of hippocampus and cortex of postmortem AD brains (Chu et al., 1987; Young, 1987) have shown a significant reduction of GABA_B_ receptors. In the same way, up-regulation of a novel non-coding RNA named 17A, RNA polymerase III-dependent embedded in the human G-protein-coupled receptor 51 gene (GPR51, GABA_B2_ receptor), affect GABA_B2_ intracellular signaling and enhances the secretion of Aβ (Massone et al., 2011). Therefore, the association between GABA_B_ receptor dysfunction and Aβ-pathological metabolism is credible in AD.

On the other hand, the expression of GABA_B_ receptors subunits may differ in accordance with the progression of AD. An immunohistochemical study in AD hippocampus demonstrated that in early stages of neurofibrillary tangle pathology the GABA_B_ receptor subunit R1 expression could be stable or increased, and then decrease as the disease progresses (Iwakiri et al., 2005). These changes indicate that compensatory mechanisms are limited and the dysfunction of hippocampal inhibitory circuitry could involve GABA_B_ receptors.

From a therapeutic point of view, some studies suggest that GABA_B_ antagonists are more likely to have neuroprotective effects than agonists (Lafon-Cazal et al., 1999; Bowery, 2006). In fact, GABA_B_ receptor antagonist may increase the expression of both neurotrophins nerve growth factor (NGF) and brain-derived neurotrophic factor (BDNF) in rat hippocampus (Heese et al., 2000). Similarly, GABA_B_ receptor agonist failed to inhibit Aβ-induced neuronal death (Lee et al., 2005; Marcade et al., 2008), while GABA_B_ antagonist may improve cognitive performance in several animal models (Farr et al., 2000; Genkova-Papazova et al., 2000). New GABA_B_ receptor antagonists have been developed for numerous *in vitro* and *in vivo* studies (Froestl, 2010). However, there is no clear evidence for its application in AD or any other cognitive dysfunction. Finally, high doses of GABA_B_ receptor antagonists as well as GABA_B_ receptor knock-out interrupt hippocampal and cortical oscillations leading to epileptiform activity and spontaneous seizures, respectively (Vergnes et al., 1997; Prosser et al., 2001; Schuler et al., 2001; Leung et al., 2005). In this sense, GABA_B_ receptors modulation must have an important function in order to reach both, an effective neuroprotection and avoid epileptiform activity.

### Aβ AND GirK CHANNELS

GABA_B_ receptors and GirK channels are coupled and co-expressed in the postsynaptic membrane of pyramidal neurons in the hippocampus (Luscher et al., 1997; Kulik et al., 2003; Lujan et al., 2009) conforming an oligomeric stable molecular complex (Lujan et al., 2009; Ciruela et al., 2010). GirK channels act as key players in the control of cellular and network excitability by modulating synaptic activity in brain structures which participate in cognitive functions (Ciruela et al., 2010). GirK channels exhibit a tonic basal activity, even without receptor signaling, due to their direct binding to the G_α_ subunit of G proteins (Lujan et al., 2009). Therefore, it is plausible an Aβ-induced intracellular signaling impairment which compromises this effector system. In fact, Aβ has been shown to disrupt G protein-coupled receptors function (Thathiah and De, 2011), and compromise coupling of the receptor with G protein (Janickova et al., 2013) as well as several different secondary messenger systems (Thathiah and De, 2009; Yang et al., 2011; Fu et al., 2012; Zhang et al., 2014). In addition, we have recently showed that Aβ decrease GABA_B_ currents in CA3 pyramidal neurons, a putative mechanism of Aβ-induced synaptic dysfunction observed in the septohippocampal system, which likely occur directly on GirK channels (Nava-Mesa et al., 2013).

GirK channels activity alteration may have multiple implications for synaptic activity, neuronal network function and cognitive processes. Numerous studies have emphasized its role in several pathological processes in the central nervous system such as epilepsy, pain, addiction, Parkinson or Down syndrome (Luscher and Slesinger, 2010). Deletion studies have revealed GirK channels role in learning and memory processes. GIRK4 knock-out mice exhibited impaired performance in a spatial learning and memory task (Wickman et al., 2000). Moreover, mutations in GIRK2 subunit reduced LTP and increased LTD in hippocampus (Sago et al., 1998; Siarey et al., 1999; Wickman et al., 2000) and it is especially relevant in Down syndrome, where cerebral Aβ accumulation is greatly accelerated and leads to invariant early onset AD neuropathology as well as learning and memory impairment (Lott and Head, 2005; Moncaster et al., 2010; Cooper et al., 2012).

Since *loss-of-function* of GirK channels might take part in the mechanisms that lead to excessive neural excitability and epilepsy that can be observed in hAPP transgenic mice model (Palop et al., 2007; Palop and Mucke, 2009, 2010a,b; Mucke and Selkoe, 2012), this type of potassium channels emerge as an interesting potential target to be studied, particularly now that its crystal structure has just been resolved (Whorton and MacKinnon, 2011).

GirK channels may be activated in a G protein-independent manner by different compounds (Kobayashi et al., 1999; Lewohl et al., 1999; Yamakura et al., 2001; Yow et al., 2011) and are blocked by different types of antidepressants (Kobayashi et al., 1999). However, there is little evidence for GirK subtype-specificity or pharmacokinetic advantages of most of those compounds, as they have other primary molecular targets (Lujan et al., 2013). However, a new class of subtype-selective agonists and antagonists has been identified (Kaufmann et al., 2013; Ramos-Hunter et al., 2013; Wen et al., 2013). An example is ML297, which has been found to be a potent, effective, and selective activator of GirK channels via a G_i/o_-coupled receptor with preference for GIRK1/GIRK2 subunit combination (Days et al., 2010). This drug displays antiepileptic properties in animals models (Kaufmann et al., 2013) and might have a possible therapeutic potential for MCI or AD. As already discussed, seizures and epileptiform activity in AD subjects support the hypothesis that aberrant network activity contributes causally to synaptic and cognitive deficits (Palop and Mucke, 2009). Thus antiepileptic drugs (i.e., *levetiracetam*) might ameliorate those deficits (Sanchez et al., 2012; Vossel et al., 2013).

In summary, tonic GirK channel activity is necessary to control neuronal excitability and synaptic function. Therefore, GirK channels emerge as an interesting potential target to understand the physiopathology of the early stages of AD. A new class of selective GirK agonists with antiepileptic properties appears as a novel therapeutic tool to be tested in further studies.

## FUTURE CLINICAL DIRECTIONS

The currently available therapy for AD (memantine, acetylcholinesterase inhibitors) may slow the progression of symptoms, but there are no existing treatments that reverse or stop disease progression even though the multiple advances in clinical and basic research.

As mentioned previously, GABA_A_ receptor agonists have shown neuroprotective effects against Aβ-induced neurotoxicity in animal and *in vitro* models. Tramiprosate is an anti-amyloid compound for the treatment of AD. This drug has shown to reverse Aβ-induced synaptic plasticity dysfunction through activation of GABA_A_ receptors (Aisen et al., 2006; Gervais et al., 2007). A phase III clinical trial showed a significant reduction in the hippocampus volume loss, but non-significant reduction in cognitive impairment (Aisen et al., 2011). Apparent divergence between neuroimaging lesions and cognitive deficits make difficult to determine reliable markers of AD. Other type of GABA_A_ agonist (i.e., benzodiazepines) may have a significant role to manage psychological symptoms associated with AD. However, the undesirable side-effects associated and plausible receptor desensitization limit their chronic use (Lanctot et al., 2007). Furthermore, cognitive decline with lorazepam in individuals with higher risk for AD (carriers of APOE-epsilon4 allele, a common AD susceptibility gene) has been reported in a double-blind crossover study (Stonnington et al., 2009).

On the other hand, GABA_B_ receptor is involved in the physiopathology of AD, and then is a pharmacological potential target. In a phase II trial, the GABAB receptor antagonist SGS742 improved cognition and memory performance in MCI possibly by up-regulation of GABAB receptors (Froestl, 2010) or through specific hippocampal protein expression (Sunyer et al., 2008; John et al., 2009). Nevertheless, in a more extensive phase IIb clinical trial in subjects with mild to moderate AD (http://www.clinicaltrials.gov/ct2/show/NCT00093951), the same drug was unsuccessful (Sabbagh, 2009).

Cumulative evidence indicates that the imbalance between excitatory and inhibitory neurotransmitter systems may underline the early cognitive deficits in AD. In this sense, antiepileptic drugs (i.e., *levetiracetam* and *topiramate*) may reverse the synaptic dysfunction associated with learning and memory impairment in animal models (Sanchez et al., 2012; Shi et al., 2013). A retrospective observational study, patients with amnestic MCI or early AD, treatment with *lamotrigine* and *levetiracetam* has shown clinical benefits and good tolerability (Vossel et al., 2013). In contrast, chronic treatment with *valproate* was associated with significant toxic effects including morphological brain changes in patients with moderate AD (Tariot et al., 2011).

Several clinical studies, including meta-analysis and clinical trials (Wei et al., 2007; Alvarez et al., 2011; Allegri and Guekht, 2012) reported the cognitive benefits of a mixture of neurotrophic factors (i.e., the nootropic agent *cerebrolysin*) as a therapeutical alternative for AD. In fact, those neurotrophic peptides can act as a neuroprotective agent or by synergically enhancing the effects of cholinesterase inhibitors (Alvarez et al., 2011). Finally, *cerebrolysin* may also regulate synaptic activity via presynaptic GABA_B_ receptors on hippocampus (Xiong et al., 1996) and it also could improve cognitive performance in patients with mild to moderate AD according to several clinical trials (Wei et al., 2007).

In conclusion, since cholinergic or glutamatergic treatments in AD have shown limited success, therapies combining modulators of different neurotransmission systems seem to be a more useful tool for the treatment, and overall prevention, of this dementia. Pharmacological strategies to recover the unbalance between excitatory and inhibitory neurotransmitters have to take into account the GABAergic system. In this sense, recent data suggest that GABA_B_ activity modulators which may control the neuronal excitability, as well as neurotrophic factors, are very interesting targets to be considered for further studies.

## CONCLUDING REMARKS

**FIGURE 1:**
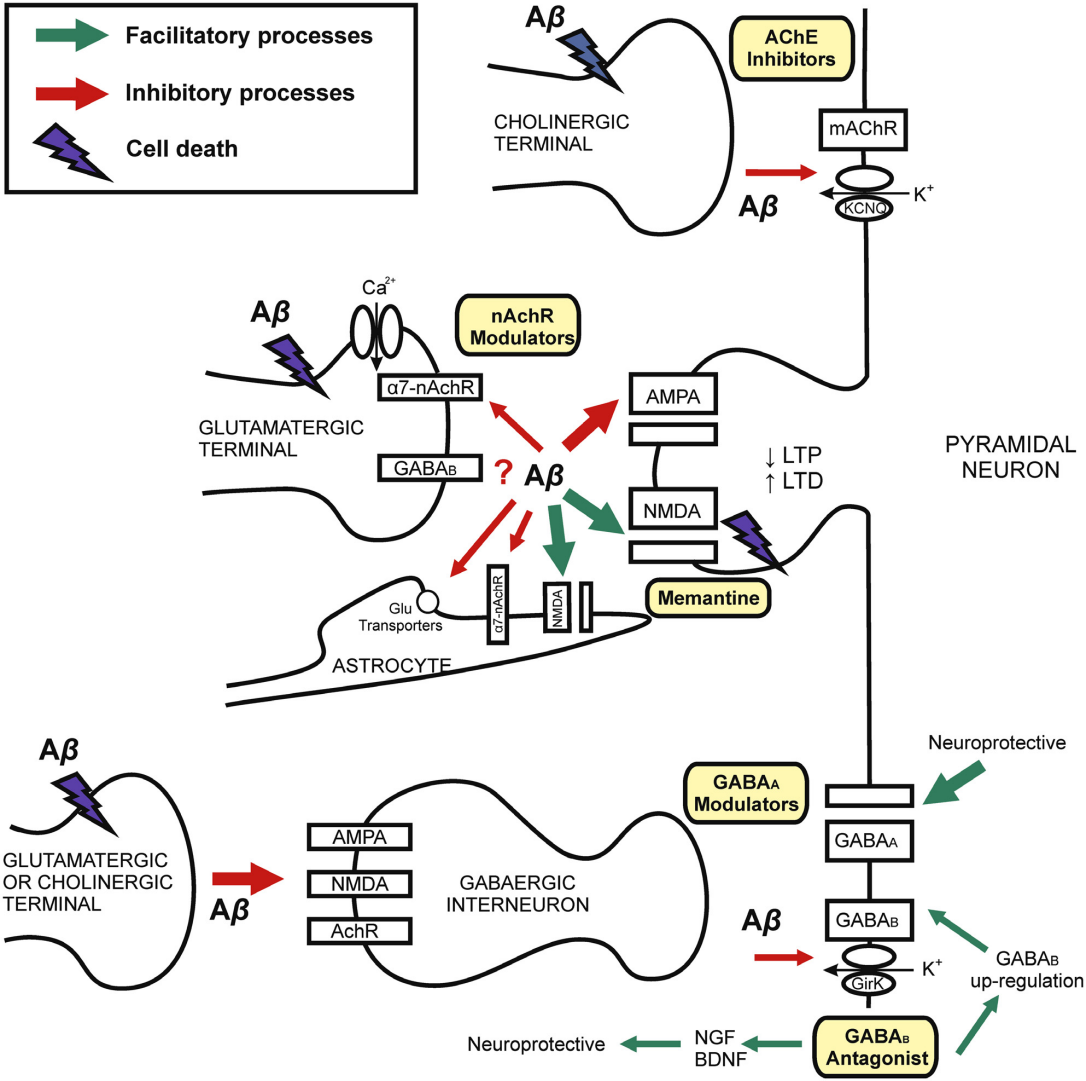
**Mechanisms of synaptic dysfunction in Alzheimer’s disease.** Schematic drawing showing the role of Aβ in the physiopathology of AD and different targets for pharmacological modulation. AD is explained by Aβ-induced neurodegeneration, therefore with decline in synaptic terminals (left) on both principal cells and inhibitory interneurons. Chronic exposition to Aβ induces neuronal death. Synaptic loss is related with cognitive impairment in AD. Early memory decline is correlated with Aβ-synaptic dysfunction on excitatory and inhibitory receptors and neurotransmission. Current evidence for the treatment of AD involve both glutamatergic (NMDA-type glutamate receptors antagonist, i.e., *memantine*) and cholinergic (i.e., *cholinesterase inhibitors*, AChE inhibitors) transmission. The GABAergic system is a possible target for developing pharmacological interventions. GABA_B_ receptors may be blocked by Aβ. However, GABA_B_ antagonists induce GABA_B_ receptors up-regulation and neurotrophic factors expression. GABA_A_ modulation (i.e., by GABA_A_ receptor agonist and selective inverse agonist) may have nootropic and neuroprotective effects. Although GABA interneurons are spared in several AD models, the imbalance between excitatory and inhibitory neurotransmission might underlie early AD cognitive dysfunction. *Abbreviations*: Aβ, Amyloid-β; AchR, acetylcholine receptor; AChE, acetyl-cholinesterase; α7-nAChR, α7-nicotinic acetylcholine receptor; BDNF, brain-derived neurotrophic factor; GirK, G protein-gated inwardly rectifying potassium channel; KCNQ, Kv7 family of voltage-gated potassium channels; LTP, long-term potentiation; LTD, long-term depression; NGF, nerve growth factor.

It is reasonable to consider that Aβ-induced pathology on inhibitory synaptic activity might be explained by both their effects on specific inhibitory circuits or indirect effects on excitatory afferents to GABA neurons (**Figure 1**). Because of the availability of Aβ on synaptic cleft and the particular vulnerability of each neuronal circuit, some Aβ effects depend on the brain structure under study. On the other hand, there is increasing evidence to suggest that GABAergic neurons are relative spared in animal models of AD-like amyloid pathology. However, Aβ modulates inhibitory GABA activity through functional compensatory up-regulation mechanisms or neurodegeneration on cholinergic and glutamatergic neurons, which in turn innervate GABA interneurons. So GABAergic neurons are responsible for brain rhythmic activity necessary for learning and memory processing. It is necessary to note that many of the differences between studies are related to the experimental model used. The Aβ peptide fraction and also the exposure time or differences between acute, sub-acute and chronically models should be highlighted. In this manner, the effects of Aβ in the short-term tend to be more functional than structural while in the long or chronic –term effects induce activation of compensatory mechanisms that possibly involve increased expression of specific receptors subunits, which would be interesting molecular targets for drug development.

Hence, new therapeutical approaches must take into account the different drug’s action mechanisms, diverse neurotransmitters systems involved and finally, selective different cell targets in order to produce a better clinical results.

## Conflict of Interest Statement

The authors declare that the research was conducted in the absence of any commercial or financial relationships that could be construed as a potential conflict of interest.
